# Applications of Nano Hydroxyapatite as Adsorbents: A Review

**DOI:** 10.3390/nano12142324

**Published:** 2022-07-06

**Authors:** Iresha Lakmali Balasooriya, Jia Chen, Sriyani Menike Korale Gedara, Yingchao Han, Merita Nirmali Wickramaratne

**Affiliations:** 1 State Key Laboratory of Advanced Technology for Materials Synthesis and Processing, Biomedical Materials and Engineering Research Center of Hubei Province, Wuhan University of Technology, Wuhan 430070, China; iresha.balasooriya@gmail.com (I.L.B.); 265998@whut.edu.cn (J.C.); sriyanimenike@gmail.com (S.M.K.G.); 2 Foshan Xianhu Laboratory of the Advanced Energy Science and Technology Guangdong Laboratory, Xianhu Hydrogen Valley, Foshan 528200, China; 3 Faculty of Medicine, Sabaragamuwa University of Sri Lanka, Belihuloya 70140, Sri Lanka; meritanirmali@gmail.com

**Keywords:** nanohydroxyapatite, adsorption, heavy metal, radionuclide, organic pollutants, fluoride ions

## Abstract

Nano hydroxyapatite (Ca_10_(PO_4_)_6_(OH)_2_, HAp) has aroused widespread attention as a green and environmentally friendly adsorbent due to its outstanding ability in removing heavy metal ions, radio nuclides, organic pollutants and fluoride ions for wastewater treatment. The hexagonal crystal structure of HAp supports the adsorption mechanisms including ionic exchange reaction, surface complexation, the co-precipitation of new partially soluble phases and physical adsorption such as electrostatic interaction and hydrogen bonding. However, nano HAp has some drawbacks such as agglomeration and a significant pressure drop during filtration when used in powder form. Therefore, instead of using nano HAp alone, researchers have worked on modificationsand composites of nano HAp to overcome these issues and enhance the adsorption capacity. The modification of cationic doping and organic molecule grafting for nano HAp can promote the immobilization of ions and then increase adsorption capacity. Developing nano HAp composite with biopolymers such as gelatin, chitosan and chitin has proven to obtain a synergetic effect for improving the adsorption capacity of composites, in which nano HAp fixed and dispersed in polymers can playmuch more of a role for adsorption. This review summarizes the adsorption properties and adsorbent applications of nano HAp as well as the methods to enhance the adsorption capacity of nano HAp.

## 1. Introduction

Hydroxyapatite (Ca_10_(PO_4_)_6_(OH)_2_, HAp) is a major constituent of mammalian hard tissues [1] and exists in natural phosphate mineral rocks [2]. Hydroxyapatite has attracted the scientific community due to its characteristics such as biocompatibility, hydrophilicity, surface functional groups, acidity, basicity, porosity, etc. Hydroxyapatite (HAp) synthetic particles have a wide range of applications in medical, environmental remediation and industry fields. They are used in biomedical devices [3], dental implants [4], biodegradable scaffolds [5] as coatings on bone implants [6] and other types of orthopedic implants [7] (Shokri et al., 2021). HAp is also used in the development of drug delivery systems for targeted treatment [8,9,10]. It also serves as a chromatographic adsorbent and a catalyst [11,12,13,14,15].

**Figure 1:**
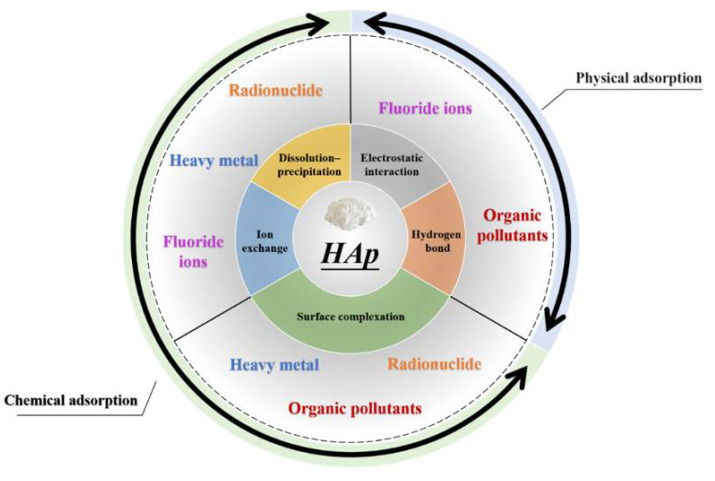
Adsorption mechanism of hydroxyapatite and its application.

The HAp crystal possesses a hexagonal structure with a six fold c-axis perpendicular to three equivalent a-axes at angles of 120° to each other. In HAp, the OH^−^ ions are aligned in columns parallel to the c-axis, along with Ca^2+^ and PO_4_^3^^−^ ions [16]. All these ions/groups and their special properties such as flexible substitutability in HAp support the adsorption mechanisms in different methods. Many investigators have reported that the sorption by HAp is taking place by ionic exchange reaction, surface complexation, the co-precipitation of new partially soluble phases and physical adsorption such as electrostatic interaction and hydrogen bonding (Figure 1).

The pathway of ionic exchange is related to the HAp properties. Ions that existed in the crystal lattice (Ca^2+^, OH^−^) can be replaced by foreign ions. Ca^2+^ in HAp can be substituted by divalent cations such as Pb^2+^, Cu^2+^, Cd^2+^ and Sr^2+^ [17]. For example, strontiapatite (Sr_10_(PO_4_)_6_(OH)_2_), formed by a substitution of calcium by strontium, is almost 107 times less soluble than hydroxyapatite [18]. Strontium substitution in natural apatites is as high as 11% [19]. The group of OH^-^ in HAp can take place by F^−^, so it can also remove halogen ions. Similarly, radio nuclides intend to be absorbed by HAp via the mechanism of ions exchange. The dynamic process of HAp dissolution–precipitation can provide PO_4_^3-^ ions and OH^-^ ions for removing foreign ions, metal ions and radionuclide and halogen ions, which is considered an important mechanism. The surface complexation mechanism, which takes an important place in the adsorption process, is dominated by the phosphate, calcium and hydroxyl groups [20]. Due to the unique surface effects of nanoparticles, there is anactive adsorption site in the HAp surface. Calcium ions exposed to the surface exhibit positively charged, easy sorption and negatively charged groups such as carboxylic acid or phosphate. The carboxyl in HAp surface possesses negative charges, forming the adsorption of cation adsorption sites. Therefore, by means of complexation the absorption of heavy metal, radial ions and organic pollutants can be achieved. In addition, substances opposite to the charge of the adsorption site can be adsorbed by HAp via electrostatic interaction belonging to physical adsorption. Hydrogen bonds play an important role in the adsorption of specific substances. The OH^-^ is located at the corners of the hydroxyapatite cell. When exposed to the crystal surface, it possibly forms a hydrogen bond with the ion to be adsorbed, which can be used in removing halogen ions and organic pollutants [21]. Compared with the larger scales such asmicro and centi, nano HAp exhibits excellent efficiency as an absorbent material due to its specific properties, such as a small size, a highly specific surface area and a more active site. The highly specific surface area takes a high surface energy, which means the process of ionic exchange surface and co-precipitation are promoted. Meanwhile, a more active site means atoms are exposed on the surface. It can form bonding with foreign substances when it is in contact with the external environment. The application of nano HAp as an adsorbent has been studied by many researchers recently. This paper reviews the up-to-date status of the applications of nano HAp as adsorbents for heavy metal ions, radionuclides, organic pollutants and fluoride ions in aqueous solutions.

## 2. Adsorption of Heavy Metals from Aqueous Solutions

With the rapid development of industries, wastewater containing heavy metals is increasingly discharged into the environment. As a consequence, heavy metal pollution has become one of the most serious environmental problems today [22]. This kind of pollution is concealed, persistent and permanent [23]. Additionally, it degrades the quality of the water and threatens the health and safety of animals and human beings by means of the food chain [23]. Therefore, it is important to improve and instigate innovative technologies for treating wastewater containing heavy metal. At present, the adsorption method is recognized as an economical and effective method for treating heavy metal wastewater. It has the following advantages: (1) The operation and design of the adsorption process are flexible, and it is suitable for efficient adsorption in most cases. (2) Adsorption materials can be reused through desorption. Many studies have recommended nano HAp as a suitable adsorbent for removing heavy metal ions such as Cd^2+^ [24,25,26,27,28,29], Co^2+^ [30,31] (Cr^6+^ [32,33], Cu^2+^ [34,35,36,37], Fe^3+^ [38]), Hg^2+^ [39], Ni^2+^ [40,41,42], Pb^2+^ [43,44,45], Zn^2+^ [46,47,48] (and As^5+^ [49] from aqueous solutions. The heavy metal ions adsorption by nano HAp is reported to be dependent on factors such as temperature, pH values. A comparison of the adsorption capacities of different adsorbents for the removal of heavy metal ions from aqueous solutions is presented in Table 1. NanoHAp and its composites display better adsorption capacities on Cd^2+^, Pb^2+^ and Zn^2+^ compared with other materials.

### 2.1. Adsorption Affinity of Different Heavy Metal Ions on Nano Hydroxyapatite

Nano HAp exhibits different removal efficiencies for different heavy metal ions. Gupta et al. reported the order of heavy metal removal efficiencies as (Pb > Co > Ni) [31]. Zou et al. discussed the adsorption and desorption process of divalent ions by HAp and found that the order of adsorption capacities was Hg^2+^> Pb^2+^> Zn^2+^> Ni^2+^> Cu^2+^> Co^2+^> Cd^2+^ ions [57]. The desorption results showed that Hg^2+^was more easily desorbed than other ions; this can be attributed to the physical adsorption [57]. The studies of Chen et al. show the order of heavy metal ions removed from aqueous solutions as Pb^2+^> Cu^2+^> Cd^2+^ and suggest that this is inversely proportional to the hydrated ionic radii as Pb^2+^ (4.01 Å) > Cu^2+^ (4.19 Å) > Cd^2+^ (4.26 Å) [24]. Mobasherpour et al. observed a heavy metal affinity to the nano HAp in a sequence of Pb^2+^>Cd^2+^> Ni^2+^ and proposed that the preference of nano HAp for a metal may be clarified by the electro negativity of the metal ions and their cation/anion state [26]. The different adsorbent efficiencies of HAp for various ions can be attributed to the different ionic radii and electronegativity of each ion.

### 2.2. Adsorption Mechanisms of Heavy Metal Ions on Nano Hydroxyapatite

**Figure 2:**
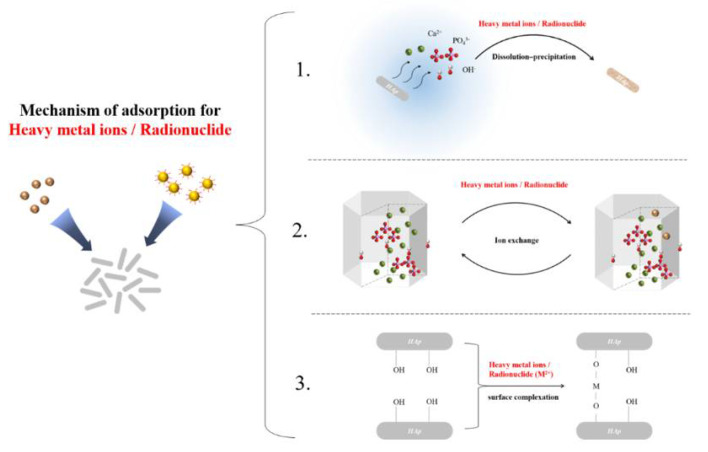
Adsorption mechanism of HAp for heavy metal ions or radionuclide.

The adsorption mechanisms of heavy metal ions on HAp are identified to be through dissolution–precipitation, ionic exchange reaction and surface complexation with calcium, phosphate and hydroxyl groups (Figure 2). After the kinetic model fitting of the adsorption process of heavy metal ions (Pt^2+^, Cu^2+^…) by HAp, it was found that the Lagergren pseudo-second order kinetic equation better describes the process [58,59]. 

Dissolution–precipitation is the main adsorption mechanism that has obtained the attention of researchers recently. As nano HAp has a greater solubility than HAp, it is dissolved easily in the aqueous solution, forming H_2_PO_4_^−^ groups which can react with heavy metal ions and form a precipitate [60].

The following reactions (Equations (1) and (2)) present the dissolution–precipitation mechanism (considering a divalent heavy metal ion M^2+^)

Dissolution:

Ca_10_(PO_4_)_6_(OH)_2 (aq)_ + 14H^+^_(aq)_→10Ca^2+^_(aq)_+ 6H_2_PO_4_^−^_(aq)_ + 2H_2_O_(l)_ (1)

10M^2+^_(aq)_ + 6H_2_PO_4_^−^_(aq)_ + 2H_2_O_(l)_→14H^+^_(aq)_ + M_10_(PO_4_)_6_(OH)_2 (S)_ (2)

Precipitation:

In the ion exchange mechanism, the heavy metal ions from the aqueous solution replace the Ca^2+^ ions of the nano HAp lattice partially, resulting in more stable heavy metal apatites [60].

The reaction below (Equation (3)) presents the ion exchange mechanism (considering a divalent heavy metal ion M^2+^)

Ca_10_(PO_4_)_6_(OH)_2_ + xM^2+^ → xCa^2+^ + Ca_(10-x)_M_x_(PO_4_)_6_(OH)_2_ (3)

Other than the two main mechanisms above, metal complexation on the nano HAp surface also takes place as a secondary mechanism (Equations (4) and(5)). The phosphate and hydroxyl groups on the HAp crystal surface facilitate the complexation mechanism [60]. 

Meanwhile, which mechanism plays a dominant role in the adsorption process depends on the nature of the adsorbed ions. For example, the adsorption process of Cd^2+^ is divided into two stages. The first stage is the complexation of ions on the HAp surface, followed by the replacement reaction between heavy metal ions and Ca ions in the HAp crystal, achieving the purpose of removing heavy metals [61]. On the other hand, the adsorption of Pb^2+^ ions is realized through the process of dissolution and precipitation [62]. Zhu et al. used layered HAp to carry out the adsorption experiment in the system of complex heavy metal ions [63]. The results showed that, in the solution of Cd^2+^, Pb^2+^, Ni^2+^, Cu^2+^ and Zn^2+^ with the same concentration, Pb is easier to be adsorbed due to it having a higher K_d_ value. Pb_10_(PO_4_)_6_(OH)_2_ is more easily formed by the reaction with phosphate dissolved from HAp [63]. 

A number of studies have found that the sorption of adsorbate on the synthesized apatites reduces the final pH to values around 6 [25,64,65]. They also found the total quantities of the displaced H^+^ are comparable to the initial metal ion concentrations in the solution. This result elucidates that heavy metal sorption affects the proton discharge from the HAp surface ≡POH sites into the aqueous solution [25].

Considering a divalent heavy metal ion M^2+^,

HAp–OH_(aq)_ + M ^2+^_(aq)_↔HAp–O–M^+^_(aq)_ + H^+^_(aq)_ (4)

2HAp–OH_(aq)_ + M ^2+^_(aq)_↔(HAp–O)_2_ M_(aq)_ + 2H^+^_(aq)_ (5)

### 2.3. Factors Affecting the Adsorption of Heavy Metal Ions on Nano hydroxyapatite

#### 2.3.1. Effect of pH

The pH level of the solution has a great influence on the surface charge of the adsorbent, the ion exchange processes and the solution chemistry of the metal ions. Previous studies have demonstrated that the adsorption capacity of HAp on heavy metal ions increases with an increase in pH in an acidic medium. Proton-competitive sorption reactions can explain this phenomenon. At lower pH values (2~6), H^+^ ions compete with metal ions for surface binding sites of nano HAp, resulting in a lower adsorption rate than at higher pH values. At higher initial pH values (6~11), the presence of the H^+^ ion in the solution is decreased, and the surface of the adsorbent is also deprotonated, increasing the metal ions adsorption [27,31,36,38,45]. However, a slight decline in sorption capacity was observed at an alkaline medium. This decline in sorption capacity in an alkaline medium was attributed to the precipitation of heavy metal hydroxides [27,31,36,38,45].

#### 2.3.2. Effect of Adsorbent Dosage

Several studies have reported the importance of sorbent dosage because it determines the sorbent capacity for a certain initial solute concentration. Elkady et al. observed a rapid increase in Cd^2+^ removal efficiency while increasing the HAp amount from 0.1 to 0.5 g, with a slight increase above 0.5 g [25]. This could be explained by the increase in sorption sites with increasing HAp amounts. Elkady et al. also observed a decrease in the amount of cadmium removed, per gram of HAP [25]. With the increase in the HAp amount from 0.1 to 1.5 g, they further clarified it with the unreacted adsorbent sites on HAp that existed during the sorption process. In the experiment of Cu^2+^ adsorption using the composite of HAp/biochar nanocomposite, Jung et al. observed an increment in the percentage removal of Cu(II) from 26.87% to 96.02% as the adsorbent dosage increased from 0.01 to 0.04 g, then reaching a constant of 100% with further increases in the dosage [66]. This was explained by the greater availability of binding sites. However, they observed the lowest adsorption capacity of 33.33 mg/g with an adsorbent dosage of 0.06 g which may be attributed to the reduction of adsorption sites caused by material agglomeration. In addition, for the low concentration of metal ions, the adsorption sites could not reach the over-saturation point due to the excessive concentration of adsorbents, so it is critical to select the appropriate amount of adsorbent [66].

#### 2.3.3. Effect of the Initial Heavy Metal Ion Concentration

Many researchers have examined the effect of the initial heavy metal ion concentration on the adsorption capacity. Elkady et al. observed an immediate reduction of adsorption with a fixed amount of HAp and suggested it was due to the deficiency of the available active sites of HAp required for the reaction [25]. They also observed an increase in the sorption capacities of nano HAp with an increasing cadmium concentration. This was possibly due to the increase in mass transfer driving force, which increases the rate of cadmium ions passing from the solution to the nano HAp particle surface [25]. (Elkady et al., 2011).

#### 2.3.4. Effect of Contact Time

Evidence from the study by Elkady et al. suggests that the cadmium sorption process is rapid at the beginning (in the first 60 min) and then becomes slow [25]. This effect was explained by Elkady et al. using the larger surface area of the sorbent available for the Cd^2+^ sorption at the beginning, resulting in a rapid process [25]. Later, the process becomes slow as a result of the sudden exhaustion of the sorption sites by cadmium ions. Mortada et al. also proposed a similar conclusion: the adsorption rate of hydroxyapatite nanorods on heavy metals gradually slowed down with the extension of time and reached the platform in 30 min, indicating that this nanorod material has a good adsorption performance [67].

#### 2.3.5. Effect of Solution Temperature

The solution temperature affects both the rate and extent of heavy metal sorption. The temperature dependency of the sorption process also offers evidence of potential sorbate–sorbent interaction. Elkady et al. examined the percentage of cadmium sorption by nano HAp with increasing temperatures [25]. Their results showed an increasing trend of sorption percentage with increasing temperatures. Elkady et al. proposed that the Cd^2+^ adsorption is stimulated by the temperature and also by the diffusion of Cd^2+^ through HAp particles [25].

#### 2.3.6. Effect of Agitation Speed

The agitation affects the distribution of the solute in the solution as well as the formation of the external boundary film. Elkady et al. observed a relationship between the agitation speed and the percentage of cadmium removal for values between 0 and 500 rpm [25]. Their results elucidated the effect of external diffusion on the sorption kinetic mechanism. An increase in the agitation speed reduces the resistance of the boundary layer to mass transfer in the solution and increases the kinetic energy of hydrated ions. However, above 500 rpm, the cadmium removal percentage decreased, possibly as a result of an increase in the desorption tendency of adsorbate ions or the similar speed of adsorbent particles and adsorbate ions [25].

#### 2.3.7. Effect of Foreign Ion

In practical applications, foreign ions are often involved in most conditions, and the interaction of these ions with the HAp surface affects the adsorption process of heavy metals in the solution. Jung et al. tested the adsorption capacity of HAp in the presence of Na^+^, NO_3_^−^, ClO_4_^−^ and Cl^−^ in the solution and found that foreign ions had no obvious effect on the adsorption process at pH=5.8.When it is taken to pH=4.0, with the increase in ionic strength, the adsorption capacity of copper ions is improved, especially NaCl [66]. A possible reason for this is that the anion acts as a bridge to form HAp≡Cl-Cu, which further shows good adsorption performance at high ion concentrations [66]. 

### 2.4. Multiple Metal Solute System

The metal ion adsorption of an aqueous solution is intensely influenced by the competition between the metal ions to occupy the limited sites. Consequently, the removal efficiency of the adsorbent for the interested metals will decrease. Hence, understanding the overall heavy metal adsorption capacity of nano HAp, particularly in multiple metal solute systems, is important.

Chen et al. investigated the efficiency of nano hydroxyapatite on adsorbing aqueous Cd, Pb and Cu [24]. The results indicated a higher potential of nano HAp to adsorb aqueous Pb than other metals. Chen et al. developed a selectivity coefficient measurement and an isotherm equation, which were used to evaluate the competitive adsorption of the metals on nano HAp in multiple metal systems [24]. The results also revealed that the adsorption capability of nano HAp for individual metal ions was reduced by the competitive adsorption of multiple metals on nano HAp. Chen et al. suggested that the level of decrease was influenced by the adsorption affinity of nano HAp to the other metal ions existing in a single-metal system (i.e., Pb^2+^ > Cu^2+^ > Cd^2+^) [24]. Although the adsorption of aqueous metal ions depends on many factors, this study found that the ratios of metal ions displaced by other competitive metal ions were moderately consistent over a wide concentration range in multiple metal systems.

The OH^−^ and PO_4_^3−^ groups in HAp are hard Lewis bases, whereas Pb^2+^ is a hard Lewis acid. Cd^2+^ and Ni^2+^ are considered soft Lewis acids. The greater affinity of Pb compared to Cd and Ni towards HAp could be a result of that. As the electronegativity of Pb is greater than Cd and Ni, it results in a greater affinity in the electrostatic and inner-sphere surface complexation reactions. Compared to cations with larger ionic radii, the cations with ionic radii smaller than Ca^2+^ (0.099 nm) have a lesser opportunity to incorporate into a HAp structure [68]. Hence, the precipitation of larger cations such as Pb^2+^ (0.118 nm) and Cd^2+^ (0.097 nm) is favorable over the precipitation of smaller cations such asNi^2+^ (0.072 nm) and Ca^2+^ [26]

### 2.5. Enhancing the Heavy Metal Ion Adsorption Capacity of Nano Hydroxyapatite

Nanohydroxyapatite (HAp) can be used as an environmentally friendly adsorbent of heavy metal ions. However, the agglomeration and precipitation of nano HAp will result in the loss of adsorption capacity to heavy metal ions. Guo et al. suggested an alternative to inhibit the agglomeration of nano HAp and to easily separate the material from the aqueous solution after the adsorption of heavy metal ions by dispersing and fixing nano HAp in a polymer [60]. Guo et al. proposed sodium alginate as a suitable candidate polymer that can easily exchange its Na^+^ ion with heavy metal ions to form gels [60]. They expected to fix HAp particles with sodium alginate and predicted the HAp-alginate composite to increase the adsorption capacity and removal efficiency with the integrated function of both components. Guo et al. suggested that nano HAp can be used as an adsorbent for heavy metal ions with alginate fixation to separate from the aqueous solution [60].

Although HAp has a good adsorption capacity, it creates a pressure drop during the field applications due to its powder form. To overcome this, Gopalakannan et al. suggested embedding HAp in a polymeric matrix, producing a hybrid composite [33]. Gopalakannan et al. investigated the Cr(VI) adsorption capacity of magnetic particles reinforced nanohydroxyapatite/gelatin composite, and they expected the HAp/ polymer hybrid composite to show excellent mechanical strength, a high sorption capacity and a higher specific surface area [33]. Their results indicated that the synthesized magnetic biocomposites exhibited a higher chromium adsorption capacity compared to nano HAp alone. As the amine group of gelatin and Lewis acid metal ions (Fe^3+^ and Ca^2+^) of the adsorbent easily protonate, creating a positive surface, negatively charged chromate anions are attracted by electrostatic attraction with surface complexation. Gopalakannan et al. also investigated the sorption capacity of insitu and hydrothermal synthesized adsorbents and observed that the adsorbents synthesized in the hydrothermal method possess a greater adsorption capacity than those in the insitu method, as the former have a high surface area, pore width and pore volume [33].

Gandhi et al. synthesized a chitin/chitosan nano HAp composite for copper (II) removal [36]. They expected that the high metal ion adsorption potential of chitin and chitosan combined with the adsorption ability will result in a higher adsorption capacity [36]. The results indicated the sorption capacities of the adsorbents in a sequence of nano HAp < nano HAp/chitin composite < nano HAp/chitosan composite [36]. Kousalya et al. examined the adsorption capacity of nano HAp/chitin and nano HAp/chitosan composites, which could possess higher mechanical strength, biocompatibility and biodegradability than the components alone [38]. The sorption mechanism of Fe(III) by nano HAp and the two composites was dominated by adsorption and ion exchange [38]. Fe(III) sorption by chelation was also observed in nano HAp/chitin and nano HAp/chitosan composites as a result of the lone pair of electrons from nitrogen, which has also taken place as a result of amino and hydroxyl groups in the composites [38]. Therefore, Kousalya et al. observed a high increase in the sorption capacity in nano HAp/chitin and nano HAp/chitosan composites compared to nano HAp alone due to the overall effect of chitin with nano HAp and chitosan with nano HAp [38].

Gupta et al. investigated the adsorption capacity of HAp/chitosan composite on the removal of Pb^2+^, Co^2+^ and Ni^2+^ from an aqueous solution [31]. They identified that chitosan binds HAp and forms aggregates to prevent the dispersion of HAp in an aqueous solution, which results in the convenience of recovering HAp. Similarly to pure HAp, HAp/chitosan composite also showed the order of removal efficiency as Pb > Co > Ni [31].

Salah et al. found that the sorption capacity of nano hydroxyapatite/chitosan composite (122 mg/g) to Cd^2+^ was greater than nano HAp alone(92 mg/g) [28]. They suggested the sorption capacity could be increased by increasing the initial Cd^2+^ concentration and also the nano HAp/Cd^2+^ mass ratio. Moreover, they identified that the regeneration capacity of the sorbent was satisfactory [28].

In addition to compounding HAp with other materials, the surface modification of HAp can be also an effective method to remove heavy metals. Oulguidoum et al. grafted 1, 3-benzenedisulfonate on a HAp surface and then carried out adsorption experiments on Pb^2+^, Zn^2+^ and Cd^2+^ ions [69]. The results indicated that, compared with the blank control group, the adsorption ability of the materials on these ions had increased significantly, which was attributed to the enhancement of the metal immobilization by the grafted groups [69]. Moreover, the grafted material still showed good adsorption performance after three cycles of adsorption–desorption, indicating the reversibility of adsorption at the solid surface sites [69].

## 3. Adsorption of Radionuclides from Aqueous Solutions

Radioactive metals are considered hazardous contaminants in the environment due to their radioactivity, toxicity and potential to be carcinogenic to humans. Hence, it is very important to select an appropriate and effective method to remove radioactive metals from water. The present methods for radioactive metal (i.e., U, Sr, Co, etc.) removal from nuclear wastewaters include precipitation, reverse osmosis, ion exchange, adsorption and ultrafiltration [70]. Most of these methods are expensive and involve high maintenance. As the adsorption process is a simple and low-cost process, many researchers have investigated the use of adsorbents. Hydroxyapatite [71,72,73,74,75,76,77,78,79,80,81], aluminum oxide [82], silicon dioxide [83], hematite [84], akageneite [85,86] zeolite [87], activated carbon [88] and titanium [89] are some of the adsorbents previously studied. HAp has been widely studied for its application as an efficient radioactive ion adsorbent. It has been recommended for the adsorption of radionuclides such as UO_2_^2+^ [73,74,77], Sr^2+^ [71,80] and Co^2+^ [90] by many researchers. A comparison of the adsorption capacities of several adsorbents for the removal of radionuclides from aqueous solutions is presented in Table 2. Compared with other absorbents, nano HAp has a good absorption capacity in U.

### 3.1. Adsorption Mechanisms of Radionuclides on Nano Hydroxyapatite

Two promising reaction mechanisms have been suggested for the reaction of HAp with uranium. They are the dissolution of HAp followed by the precipitation of chernikovite and autunite [73,94] by surface adsorption or complexation [95]. According to previous studies, the precipitation of chernikovite and autunite can scarcely occur with a low uranium concentration (4700 ppm) in the removal process. Therefore, surface complexation is recognized as the main uranium removal method in low concentrations of uranium [74] (Li et al., 2012) (Figure 2).

Handley-Sidhu et al. suggested the following equations (Equations (6)–(8)) on the mechanisms of Sr^2+^ adsorption on nano HAp [71].

Surface adsorption:

OH + M^2+^→≡O−M^2+^ + H^+^ (6)

O_3_P−OH^+^ + M^2+^→≡O_3_P−O−M^2+^ + H^+^ (7)

Ion exchange:

Ca^2+^ + M^2+^ →≡M^2+^ + Ca^2+^ (8)

Further, it has proven that the Lagergren pseudo-second order kinetic equation can better match the process of radionuclide adsorption on HAp [96].

### 3.2. Factors Affecting the Adsorption of Radionuclide Ions on Nano Hydroxyapatite

#### Effect of the pH Variation

Bulk precipitation is the main mechanism of Uranium(VI) removal by HAp, and the formed precipitates intensely relate to the pH of the solution. In a solution where pH is between the mildly acidic and the moderate alkaline region, uranium is precipitated as its phosphate salts, such as (UO_2_)_3_(PO_4_)_2_ or Ca(UO_2_)_2_(PO_4_)_2_. Uranium may precipitate as calcium dioxouranium(VI) carbonate (CaUO_2_(CO_3_)_2_) or as calcium uranate (CaUO_4_ and/or Ca_3_UO_6_) in strongly alkaline regions [73]. According to Krestou et al., in the neutral pH region, the form of uranium removed from the solution was very stable but it was unstable in the high alkaline region, in which a considerable amount of uranium (VI) was redissolved in the aqueous media [73] (Krestou et al., 2004). Therefore, many studies have suggested that HAp has a strong affinity to efficiently remove U(VI) around pH 5 to 6, but U sorption is partly reversible in the high alkaline region, facilitating the remobilization of the contaminant [73,97]. 

## 4. Adsorption of Organic Pollutants from Aqueous Solutions

Researchers have examined different adsorbent materials such as activated carbon [98], biochar [99,100], natural clay [101], carbon aerogels [102], HAp, etc. for organic pollutant removal from an aqueous solution. Hydroxyapatite is a biomaterial widely used in water pollution control as a result of its excellent adsorption affinity, inexpensive nature, accessibility and environment ally friendly nature. The use of nano-sized hydroxyapatite is advantageous over normal-sized hydroxyapatite due to the high surface area and reactivity as well as the ability to disperse throughout an aqueous solution (Table 3). A comparison of the adsorption capacities of different adsorbents for the removal of organic pollutants from aqueous solutions is presented in Table 3. For all organic pollutants, nano HAp shows an excellent adsorption capacity compared to other absorbents.

### 4.1. Adsorption Mechanisms of Organic Pollutants on Nano HAp

**Figure 3:**
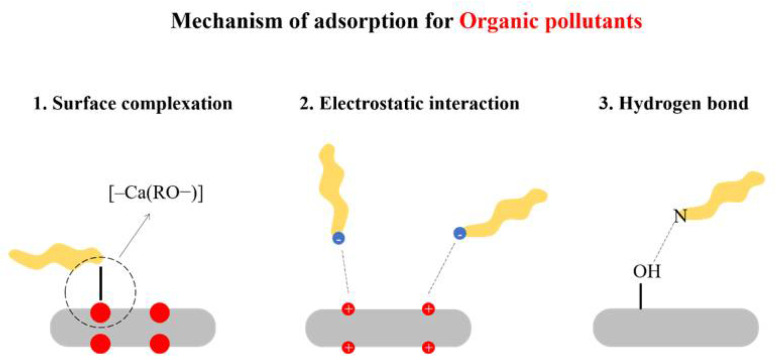
Adsorption mechanism of HAp for organic pollutants.

There are threemain adsorption mechanisms of HAp that affect organic pollutants: electrostatic attraction, surface adsorption and hydroxyl bond(Figure 3). The process of adsorption conformsmore to theLagergren pseudo-second order kinetic equation.

In the dissociation of phenolic compounds, their oxygen gains the greatest effective charge, acting as adsorption centers in the adsorption onto HAp. Molecules containing hydroxyl-groups contest with water molecules in the solution for the surface calcium ions of HAp. They are adsorbed onto HAp in an ionic form by forming [–Ca(RO−)] complexes [103].

The reactive blue 19 dye adsorption on HAp in acidic pH may be explained by the electrostatic attraction between the positively charged ≡CaOH^+^ groups on the HAp surface and the negatively charged sulfonyl (–OSO_3−_) and sulfonic (–SO_3−_) groups of the reactive blue 19 dye [107] 

The adsorption of reactive red 198 dye on HAp can be explained as follows. The dye molecules are adsorbed through the electrostatic attractions between the ionized sulphonyl groups of the dye molecule and the positively charged Ca^2+^ of the HAp surface. The dipole–dipole hydrogen bonding interaction occurs between the azoic group of the dye molecule and the hydroxyl groups of HAp, as well as the electrostatic interactions which may occur between the cationic groups of a dye such as Na^+^ with anionic groups of HAp such as OH^−^ and PO_4_^3−^ [109].

The mechanism of the HAp adsorption of methylene blue can be explained as follows: under alkaline conditions, the negative charge on the surface of hydroxyapatite and the key group of methylene blue play a dominant role. Under acidic conditions, the electrostatic effect does not exist because the surface of hydroxyapatite is positively charged, and the adsorption is achieved by the (P-OH) group of HAp-particles and the (N) of the methylene blue molecule. Therefore, the adsorption mechanism is related to the external condition [111].

### 4.2. Factors Affecting the Adsorption of Organic Pollutants on Nano Hydroxyapatite

#### 4.2.1. Effect of pH

The pH of the adsorption medium is considered the most critical parameter in the phenol adsorption process by nano HAp, because the charge of both the adsorbate and the adsorbent depends on the pH of the solution. Lin et al. studied the effect of the initial pH of the solution on the phenol adsorption onto nano HAp and found that the adsorption capacity is decreased with increasing pH up to 8.2, and then the adsorption capacity is increased by further increasing the pH to the alkaline value [103]. The maximal adsorption capacity was observed at pH 2 [103]. 

In the nitrobenzene adsorption process, the pH of the adsorption medium is the most vital parameter. Wei et al. found that the adsorption capacity of nanocrystalline HAp decreases sharply when the pH is higher than 6.0, and the maximum adsorption capacity of nanocrystalline HAp on nitrobenzene was at pH 2.0 [105].

Vasugi et al. studied the reactive red 198 dye adsorption capacity of nano HAp under different pH values and learned that nano HAp showed a better dye removal capacity in acidic pH and that the maximum dye removal capacity was observed at pH 6 [109].

Ciobanu et al. examined the removal rate of reactive blue 19 dye by nano HAp under different pH values [107]. Ciobanu et al. found that nano HAp exhibited a higher adsorption capacity in acidic pH and a lower adsorption capacity in basic pH, while the maximum adsorption was seen at pH 3 [107].

Allam et al. (2016) proposed that when the pH value in the solution is higher or lower than the HAp isoelectric point, the charge on the surface of the nanoparticles will change because the HAp isoelectric point is between 6 and 7.2, which will affect the mechanism of organic matter adsorption [111].

#### 4.2.2. Effect of Contact Time

Lin et al. studied the effect of contact time on the phenol adsorption by HAp nanopowders [103]. They found that the adsorption equilibrium of phenol was achieved after 2 h and observed no remarkable changes for longer contact times [103].

Wei et al., examined the effect of contact time on the adsorption of the nitrobenzene on nanocrystalline HAp for initial nitrobenzene concentrations of 5, 10 and 50 mg/L [105]. For the three concentrations above, the adsorption equilibrium of nitrobenzene was obtained within 1 min [105]. They observed no further changes in prolonging contact times. The equilibrium time of different initial nitrobenzene concentrations exhibited that initial nitrobenzene concentrations had little effect on the adsorption equilibrium time [105]. 

Vasugi et al. investigated the effect of contact time on the reactive red 198 dye removal percentage by HA [109]. They observed an increase in the dye removal percentage with time and found that, above 360 min, it approaches equilibrium [109] Ciobanu et al. examined the effect of contact time on the reactive blue 19 dye removal by nano HAp at an initial pH of 3 with a nano HAp dose of 2 g/L and an initial reactive blue 19 dye concentration of 65 mg/L under 20 °C temperature [107]. They observed a rapid increase in the amounts of the dye adsorbed onto nano HAp within the first 30 min, changing slightly in the subsequent 3 h when the maximum adsorption was reached [107]. Thereafter, no further adsorption occurs with a longer contact time, indicating the reaching of equilibrium [107]. 

#### 4.2.3. Effect of Adsorbent Dosage

Lin et al. investigated the effect of the nano HAp adsorbent dose on the percentage of phenol removal [103]. They identified an obvious increase in the phenol adsorption percent with increasing HAp dosages [103]. The phenol adsorption percent increased rapidly from 32.5 to 35.5% with the increase in HAp dosage from 2 to 4 g/L [103] This was attributed to the increase in the number of adsorption sites with the increase in the HAp adsorbent dosage [103]. As the HAp dosage was further increased to 12 g/L, the phenol adsorption percent increased slightly to 37.0% [103]. The results also revealed the possibility to remove phenol completely with sufficient HAp in the solution [103]. 

Wei et al. examined the effect of the adsorbent dose on the nitrobenzene removal by nano HAp over the 2.0–10 g/L range of the adsorbent, keeping all other parameters constant [105]. They observed an increase in the percent removal for nitrobenzene with the increasing adsorbent dosage [105]. A rapid increase in the nitrobenzene removal efficiency from 66.2 to 70.9% was observed with the increase in nano crystalline HAp dosage from 2 to 5 g/L [105]. This increase in the nitrobenzene removal efficiency was attributed to an increase in the adsorbent concentration, which increased the available surface area and adsorption sites [105] When the nanocrystalline HAp dosage was further increased to 10 g/L, the nitrobenzene removal efficiency increased slightly to 72.6% [105]. 

Vasugi et al. evaluated the effect of nano HAp adsorbent dosage on the percentage of reactive red 198 dye removal [109]. At a pH of 6 and under a constant adsorbate concentration of 50 mg/L, they observed an increase in the dye removal percentage with the increasing adsorbent dosage as a result of the increase in the surface area available for adsorption [109]. A maximum of 89% was attained for dye removal by nano HAp [109]. 

Ciobanu et al. studied the effect of nano HAp adsorbent dose on the reactive blue 19 dye removal [107]. The dye adsorption percent was studied with different adsorbent concentrations (1–20 g/L) under constant conditions, including an adsorbate concentration of 65 mg/L, an initial pH of 3, a contact time of 3 h and a temperature at 20 °C [107]. Ciobanu et al. found an obvious increase in the dye adsorption percent with the increasing adsorbent dosage. The maximum adsorption capacity was observed at a nano HAp adsorbent dose of 2 g/L [107].

### 4.3. Enhancing the Organic Pollutant Adsorption Capacity of Nano Hydroxyapatite

Even though the adsorption by nano HAp is recognized as a promising method for nitrobenzene removal from wastewater, there are some drawbacks of nano HAp, such as poor strength, low stability and a significant pressure drop during filtration when used in powder form [116]. To prevail over these limitations and to enhance the adsorption capacity of nano HAp, several scientists have attempted to composite nano HAp with biopolymers [104,113,117]. (Wei et al. examined the possibility of using hydroxyapatite–gelatin nanocomposite for the nitrobenzene removal from an aqueous solution [104]. The study by Wei et al. revealed that the adsorption process of the hydroxyapatite–gelatin nanocomposite was fast, and it takes only 1 min to reach a steady state [104]. Wei et al. estimated the maximum adsorption capacity to be 42.373 mg/g, which was higher than many previously reported adsorbents for nitrobenzene removal [104].

Vasugi et al. investigated the reactive red 198 dye adsorption by nano HAp and a trivalent cation (yttrium) substituted HAp (Y-HAp) [109]. The study revealed that Y-HAp showed enhanced adsorption affinity compared to the pristine HAp due to the presence of additional adsorption sites owing to the difference in the oxidation state of the substituent and its related charge balance [109].

Hou et al. prepared the composite of HAp and chitosan, which was used to adsorb Congo red dye [113]. Compared with the adsorption capacity of pure HAp (305 mg/g), the adsorption capacity of composite material increased by more than two times (769 g/mg) [113]. The characterization results show that the adsorption process not only includes electrostatic adsorption but also involves complexation, ion exchange and hydrogen bonding [113].

Guan et al. prepared polyalcohol modified HAp nanoparticles with a core-shell structure using D-Fructose-1, 6-phosphate trisodium salt octahydrate (C_6_H_11_Na_3_O_12_P_2_·8H_2_O, DFP) as a phosphorus source [118]. The material has a large surface area of 203.18 m^2^/g, so it shows good adsorption performance for organic excitements such as methyl orange and Congo red [118].

## 5. Adsorption of Fluoride Ions from Aqueous Solutions

The consumption of drinking water with higher levels of fluoride leads to serious health effects on human beings, including fluorosis. According to the World Health Organization, the maximum acceptable fluoride concentration in drinking water is 1.5 mg/L [119]. To remove excess fluoride from water, different defluoridation methods have been adopted. These methods include ion exchange [120], adsorption [121], precipitation [122] electrolysis [123], nanofiltration [124] and reverse osmosis [125].

Among the mentioned methods, adsorption is considered effective because it is an easy, versatile and cost-effective method. Researchers have investigated various adsorbent materials for fluoride removal from an aqueous solution. Calcite [126], limestone [127], nanohydroxyapatite [128,129,130,131,132,133,134], montmorillonite [135], mixed metal oxides [136], activated carbon [137], activated alumina [138], layered double hydroxides [139], clays [140] and rare earths-loaded chitosan beads [141] are some of them. Among these adsorbents, nano HAp has attracted researchers’ interest due to its chemical composition, crystal structure, excellent defluoridation capacity, low cost and availability. Several attempts have been made to composite nano HAp with polymers and also to use surface modification techniques to enhance the fluoride removal capacity of nano HAp. A comparison of the defluoridation capacities of different adsorbents including HAp for the fluoride removal from aqueous solutions is presented in Table 4. Nano HAp is a promising material for removing F^-^ compared to other materials.

### 5.1. Adsorption Mechanisms of Fluoride Ions on Nano Hydroxyapatite

**Figure 4:**
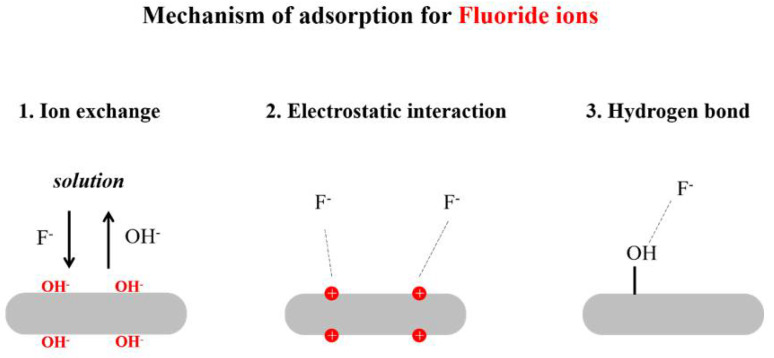
Adsorption mechanism of HAp for fluoride ions.

The mechanism of fluoride removal by nano HAp is controlled by both adsorption and ion exchange mechanisms [130,145]. In an aqueous fluoride solution, fluoride ions are adsorbed by nano HAp according to the reaction below. In addition to this, the OH^−^ ions of the n-HAp lattice are replaced by the F^−^ ions by ion exchange. (Figure 4) Based on the data of the experiment, the Lagergren pseudo-second order kinetic equation better fits its adsorption process for F^−^.

The adsorption of F^−^ ions on n-HAp could be expressed by the following reaction (Equation (9)):

Ca_10_(PO_4_)_6_(OH)_2_+nF^−^→Ca_10_(PO_4_)_6_(OH)_2_---F_n_^−^ (Hydrogen bonding) (9)

The fluoride removal of the n-HAp by ion exchange could be expressed by the following reactions (Equations (10)and(11)) [146]:

Ca_10_(PO_4_)_6_(OH)_2_+ F^−^→Ca_10_(PO_4_)_6_FOH + OH^−^ (10)

Ca_10_(PO_4_)_6_(OH)_2_+2F^−^→Ca_10_(PO_4_)_6_F_2_ + 2OH^−^ (11)

In acidic media, the nano HAp surface gains a positive charge due to the higher H^+^ concentration of the medium. Therefore, nano HAp attracts more fluoride ions in an acidic medium by electrostatic attractions, resulting in a higher defluoridation capacity at lower pH values. In alkaline pH, the nano HAp surface gains a negative charge and repels negatively charged fluoride ions, preventing the electrostatic attractions.

The fluoride removal of n-HAp in an acidic medium could be expressed by the following reaction (Equation (12)):

Ca_10_(PO_4_)_6_(OH)_2_+nF^−^→Ca_10_(PO_4_)_6_(OH^+^)_2_---F_n_^−^ (Hydrogen bonding and electrostatic attractions) (12)

### 5.2. Factors Affecting the Adsorption of Fluoride Ions on NanoHydroxyapatite

Several factors such as the solution pH, contact time, adsorbent dose and other anions in the medium affect the adsorption of fluoride ions on nano HAp.

#### 5.2.1. Effect of pH

Among the factors mentioned above, the pH value plays an important role in the adsorption of fluoride ions at the water adsorbent interface. Sundaram et al. studied the fluoride adsorption on nano HAp under different pH values from 3 to 11 at room temperature with an initial fluoride concentration of 10 mg/L [130]. The removal of fluoride ions reached a maximum value of 1845 mgF^−^ kg^−1^ at pH 3. At pH 11, the defluoridation was only 570 mgF^−^ kg^−1^. Accordingly, the defluoridation capacity increases with the decreasing pH [130]. This phenomenon is associated with the change of the adsorbent’s surface charge. In an acidic medium, the surface of the adsorbent is highly protonated, and as a result, the attractive forces between the positively charged nano HAp adsorbent surface and the negatively charged fluoride surface gradually increase with decreasing pH values, resulting in an increase in the fluoride removal capacity by nano HAp. A lower defluoridation capacity by nano HAp in an alkaline medium is attributed to the nano HAp surface gaining a negative charge in the alkaline pH, resulting in repulsion between the negatively charged nano HAp surface and fluoride.

#### 5.2.2. Effect of Contact Time

The adsorption of aqueous fluoride ions is also strongly influenced by contact time. Sundaram et al. examined the fluoride adsorption on nano HAp, varying the contact time in the range of 10–60 min with an initial fluoride concentration of 10 mg/L at room temperature [130]. (They observed that it took 30 min to reach the saturation and suggested that both ion exchange and adsorption control the fluoride sorption on nano HAp [130]. This is because, if the sorption process was only governed by the ion exchange mechanism, the saturation must have been reached very soon. As nano HAp reached saturation after 30 min, they proposed that adsorption, which is a slow process compared to ion exchange, also plays a key role in the sorption process [130]. 

#### 5.2.3. Effect of Adsorbent Dose

Sundaram et al. studied the effect of nano HAp dose on percent fluoride removal by using different dosages of nano HAp ranging from 0.1 g to 1.0 g with an initial fluoride concentration of 10 mg/L [130]. They observed an increase in the percent fluoride removal with increasing adsorbent dosages. This was attributed to the added active sites resulting from the increase in the adsorbent dosage.

Jiménez-Reyes and Solache-Ríos conducted experiments using hydroxyapatite to adsorb 5 mg/L fluoride ions [147]. It has no obvious proportional relationship between the sorbent and sorbent dosage, and the following equation is used to fit the experiment data:q_e_ =−3.3(±0.2)×(ln X)−2.2(±0.2); R^2^ = 0.996, where q_e_ is the adsorption capacity of Hap [147]. 

#### 5.2.4. Effect of Other Anions in the Medium

Co-existing anions in the aqueous solution may compete with fluoride ions for adsorption sites during defluoridation and cause a negative effect on the defluoridation capacity. Sundaram et al. investigated the effect of the co-anions including Cl^−^, NO_3_^−^ and SO_4_^2−^ HCO_3_^−^ on the defluoridation capacity of nano HAp by keeping the initial concentrations of these ions ranging from 100–500 mg/L and 10 mg/L as initial fluoride concentration at 303 K [130]. Sundaram et al, observed no significant difference in the defluoridation capacity of nano HAp in the presence of Cl^−^, NO_3_^−^ and SO_4_^2−^ ions [130]. In presence of HCO_3_^−^ ions, the defluoridation capacity considerably decreased. Sundaram et al. concluded that the decrease in the defluoridation capacity is due to the competition of HCO_3_^−^ ions with the fluoride ions [130]. In another study, the interference of bicarbonate ions was explained by the increase in the solution pH due to the release of OH^−^ ions from the NaHCO_3_ hydrolysis and their competition with fluoride ions for active sites on nano HAp [148]. 

### 5.3. Enhancing the Fluoride Ion Adsorption Capacity of NanoHydroxyapatite

Although nanohydroxyapatite has been identified as a promising defluoridating material, its use is limited. The reasons are its brittleness and the difficulty for it to be used directly in fixed bed columns because of the significant pressure drop it causes in field applications. To overcome such technological barriers and to enhance the fluoride adsorption capacity of nanohydroxyapatite, different techniques have been used.

The collective effect of biopolymer and inorganic material has the ability to increase the mechanical properties of the composite [131]. Sundaram et al. employed nano HAp/chitin composite as adsorbents for the uptake of fluoride ions and found that it possesses a higher defluoridation capacity (DC) of 2840 mgF^−^ kg^−1^ than nano HAp alone, which has a DC of 1296 mgF^−^kg^−1^ [129]. Sairam Sundaram et al. proposed that the enhancement in DC may be a result of adsorption by physical forces, biosorption by chitin and the fluoride ions trapped in fibrillar capillaries and voids of the polysaccharide complex of the chitin portion of the composite [129]. Sairam Sundaram et al. suggested nanoHAp/chitin composite as a promising candidate for defluoridation [129]. Pandi and Viswanathan found that the nanoHAp/Alginate composite has an enhanced DC of 3870 mg F^−^/kg in comparison to nano HAp, which possessesa DC of 1296 mg F^−^/kg [131]. Pandi and Viswanathan synthesized nano HAp-incorporated gelatin biocomposite (n-HAp@Gel) by the in situ coprecipitation method and investigated the removal of fluoride from an aqueous solution [132]. They observed an enhanced DC in the n-HAp@Gel biocomposite, which is 4157 mgF^−^/kg compared to that of nano Hap [132]. Pandi and Viswanathan reported that in the fluoride removal mechanism, the fluoride ions are attracted by the Ca^2+^ in the gelatin polymatrix via electrostatic attraction [132]. In addition, the OH^−^ ions of the n-HAp lattice are replaced by F^−^ ions by means of ion exchange [132]. However, in the n-HAp@Gel composite, the neutralization of Ca^2+^ does not occur and, consecutively, permits the entrapping of fluoride ions from the solution as a result of the electrostatic adsorption as well as the strong Lewis acid–base interaction [132]. Pandi and Viswanathan revealed that the n-HAp@Gel composite can be successfully utilized for the adsorption of fluoride [132].

The excepted compound with organic compounds, adding inorganic substances to improve the adsorption capacity, is also a potential method. Pandi and Viswanathan prepared Fe_3_O_4_ /HAp /Chitosan as adsorbents to remove fluoride ions from the solution [134]. Compared with pure hydroxyapatite, the composite can reach adsorption saturation in a shorter time (20 min). Pandi and Viswanathan believed that, in addition to the ion exchange of F^−^ and OH^−^ and the electrostatic adsorption of Ca^2+^ and F^−^, the adsorption mechanism also involved the complexation of Fe^3+^ and F^−^ [134]. Moreover, due to the magnetism of materials, it can offer the possibility of easier separation, washing and reuse [134]. 

Cationic doping can change the microstructure and properties of the crystal. This method is considered to improve the defluorination ability of hydroxyapatite [149,150]. Chen et al. prepared HAp doped with Al^3+^, Mg^2+^ and La^3+^ to adsorb fluoride ions [149]. The results show that the doped ions can enhance the hydroxyl content of the crystal, thus providing more active sites for ion exchange, further improving the performance of fluoride ion adsorption [149]. Because the trivalent cations of Al^3+^ and La^3+^ are greater than the two valence cations of Mg^2+^, the surface part has a more positive charge, which makes its adsorption ability stronger [149]. The doping ion radius does not have an effect on the adsorption performance [149]. At the same time, Chen et al. proposed that the mechanism of fluoride ion adsorption can be divided into two stages: first, fluoride ions reach the surface of the crystal through the physical adsorption; secondly, they exchange with OH^-^ in the hydroxyapatite crystal [149]. 

Surface modification technology has been verified to be efficient and effective in enhancing the adsorption capacities of adsorbents. Muthu Prabhu and Meenakshi investigated the fluoride adsorption capacity of nano HAp surface-modified by cationic surfactants, namely, cetyltrimethyl ammonium bromide (CTAB), hexadecylpyridinium chloride (HDPC) and dodecyltrimethyl ammonium bromide (DTAB) [148]. The amine salt and quaternary ammonium saltcan offer more positive sites to the adsorbents to which they are added. Even though nano HAp has a few positively charged functional groups on the surface, the cationic surfactants CTA^+^, DTA^+^ and HDP^+^ added more positive adsorption sites to it. When a positively charged surface such as HAp is modified with a cationic surfactant, the hydrophilic groups orient away from the similarly charged substrate and towards the solution. By these means, the hydrophilic character of the adsorbent is increased, and, thus, more fluoride ions are attracted. The fluoride adsorption capacities of the three adsorbents followed the order: CTAB-HAp > DTAB-HAp > HDPC-HAp [148]. The low adsorption capacity of HDPC-HAp is a result of its pyridinium cationic head groups, which are bulkier compared to CTAB and DTAB [148] The access of fluoride to the tertiary cationic part in HDPC was blocked by the electron-rich pyridinium group, reducing the fluoride adsorption capacity compared to CTAB-HAp and DTAB-HAp [148]. Muthu Prabhu and Meenakshi, concluded that the cationic surfactant-coated HAp exhibits an excellent fluoride removal capacity over bare HAp because of the introduction of a more positive charge, especially CTAB-modified HAp compared to DTAB-HAp, HDPC-HAp powder [148]. They suggested that CTAB-HAp powder can be an auspicious defluoridating agent [148]. 

## 6. Conclusions and Future Work

The review presents the adsorption applications of nano HAp and its composites in the removal of heavy metal ions, radionuclides, organic pollutants and fluoride ions from wastewater. Nano HAp adsorbent shows a high and constant removing ability in an alkaline medium(pH 7–10)for metal ions and radionuclides. A higher alkaline region is not beneficial for the adsorbent of nano HAp; however, the acidic condition facilitates the removing of organic pollutants and fluoride ions. Usually, the sorbent capacity of nano HAp increases with a rising adsorbent dosage, but too of a high dosage cannot lead to an improvement in capacity due to the agglomeration of nano HAp. Nano HAp has the advantage of removing low concentrations, especially trace pollutants. Nano HAp shows a quick adsorption, and the adsorption rate gradually declines with the extension of contact time and reaches the platform in 30–60 min for ions; it takes more time (2–6 h) to reach the adsorption equilibrium for the majority of organic pollutants. In the future, nano HAp and its composites may be advantageous as adsorbents due to their high adsorption capacities compared to other adsorbents. Furthermore, many researchers are branching out with the modification of nano HAp, using techniques such as surface modification. However, the brittleness of HAp limits its applications. Moreover, nano HAp powder causes excessive pressure drops in field applications and therefore cannot be directly used in fixed bed columns. To overcome these technological problems, the use of composites or modification for HAp has been extensively studied. Nano-sized hydroxyapatite has its disadvantage in that the small particle size (nano-scale) makes it very difficult to separate from an aqueous solution. However magnetic adsorbents can avoid this problem, as they can be separated easily from the solution by using an external magnetic field. Hence, combining both nanotechnology and the magnetic separation technique in developing nano HAp based adsorbents would overcome the above drawback. On the other hand, the separation of nanoparticles from aqueous solutions limits the application of HAp with magnetic materials such that it only can be used in pollution removing at small volumes, such as a bottle. For its application in rivers and lakes, HAp manufactured in a layer or block is necessary. Sintering is a common forming method which can efficiently process large-scale production. However, it is difficult to preserve the excellent properties of nanoparticles. So, composites of HAp and polymers are considered as a promising method for forming. The high dispersion of nano HAp in composites should be focused on. It can be concluded that HAp is a promising material as an adsorbent for heavy metal ions, radionuclides, organic pollutants and fluoride ions in wastewater treatment. More research efforts on the practical applications development of these fascinating materials are highly recommended for the future.

## Figures and Tables

**Table 1:** Comparison of the adsorption capacities of nano HAp and its composites with other absorbents on heavy metal ions.

Adsorbates	Adsorbents	Adsorption Capacity (mg/g)	Refs.
Cd(II)	Synthetic nano crystallite hydroxyapatite	142.857	[27]
	Nanohydroxyapatite/chitosan composite	243.90	[28]
	Hydroxyapatite-magnetite-bentonite composite	310.36	[29]
	Unmodified nanodiamond (ND)	40.9	[50]
	Oxidized nanodiamond (Ox-ND-1.5)	52.9	[50]
	Oxidized nanodiamond (Ox-ND-3)	67.9	[50]
Co(II)	Hydroxyapatite/chitosan composite	10.63	[31]
	Calcined *Umbonium vestiariums* nail shell (CUVS)	93.46	[51]
Cr(VI)	Nano Hydroxyapatite	2.18	[32]
	Fe_3_O_4_@n-HApGel composite (In-situ)	18.45	[33]
	Fe_3_O_4_@n-HApGel composite (Hydro)	27.06	[33]
	Unmodified nanodiamond (ND)	33.6	[50]
	Oxidized nanodiamond (Ox-ND-1.5)	44.1	[32]
	Oxidized nanodiamond (Ox-ND-3)	55.9	[50]
Cu(II)	n-HAp	4.7	[36]
	n-HAp/chitin composite	5.4	[36]
	n-HAp/chitosan composite	6.2	[36]
	Unmodified nanodiamond (ND)	25.2	[50]
	Oxidized nanodiamond (Ox-ND-1.5)	30.5	[50]
	Oxidized nanodiamond (Ox-ND-3)	44.5	[50]
	Neem bark nanoporous adsorbent (nANB)	21.23	[52]
Fe(III)	n-HAp	4.238	[38]
	n-HAp/chitin composite	5.800	[38]
	n-HAp/chitosan composite	6.753	[38]
	Unmodified nanodiamond (ND)	26.8	[50]
	Oxidized nanodiamond (Ox-ND-1.5)	31.3	[50]
	Oxidized nanodiamond (Ox-ND-3)	45.7	[50]
Hg(II)	Chitosan/nanohydroxyapatite composite	111.6	[39]
	Magnetic mesoporous silica/chitosan (MMS/CS)	478.47	[53]
	Exfoliated graphene oxide–L-cystine	79.36	[54]
Ni(II)	Hydroxyapatite/chitosan composite	8.54	[31]
	Nanocrystalline calcium hydroxyapatite	46.17	[41]
	Activated carbon (AC) prepared from waste Parthenium	54.35	[55]
Pb(II)	Hydroxyapatite/chitosan composite	12.04	[31]
	Nano hydroxyapatite	357.14	[44]
	Nanohydroxyapatite–alginate composite adsorbents	270.3	[45]
	Mg_2_Al-LS-LDH composite	∼123	[56]
Zn(II)	Nano hydroxyapatite	57.504	[46]
	Neem bark nanoporous adsorbent (nANB)	11.904	[52]

**Table 2:** Comparison of the adsorption capacities of nano HAp and its composites with other absorbents on Radionuclide.

Adsorbates	Adsorbents	(mg/g)	Refs.
Sr (II)	Nanocrystalline bio-hydroxyapatite	5.35	[71]
	Commercial-hydroxyapatite	0.76	[71]
	[MeNH_3_]5.5[Me_2_NH_2_]0.5In10S18·7H_2_O	151.2	[91]
U (VI)	In situ-grown nanohydroxyapatite on magnetic Ca Al-layered double hydroxides	261.1	[73]
	Hydroxyapatite from bones combustion	20	[74]
	Bio-hydroxyapatite (Bio-HAP600)	384.6	[92]
	γ-Fe_2_O_3_	87.35	[93]

**Table 3:** Comparison of the adsorption capacities of nano HAp and its composites with other absorbents on organic pollutants.

Adsorbates	Adsorbents	Q (mg/g)	Conditions	Refs.
Phenol	Hydroxyapatite nanopowders	10.33	333 K, pH 6.4	[103]
	Natural clay	15	298 K, pH 5	[101]
Nitrobenzene	Hydroxyapatite–gelatin nanocomposite	42.373		[104]
	Nanocrystalline hydroxyapatite	8.993	298 K	[105]
	Hydrophobic cotton fibers adsorbent	16.85	293 K	[106]
Reactive Blue 19 dye	Uncalcined nanohydroxyapatite	90.09	293 K, pH 3	[107]
	Calcined nanohydroxyapatite	74.97	293 K, pH 3	[107]
	Chitosan coated magnetic hydroxyapatite	26.4	pH 5	[108]
Reactive red 198 dye	HA	21.5		[109]
	Yttrium substituted HA	25.3		[109]
	Native pretreated dried *Potamogeton crispus*	14.3		[110]
	Acid pretreated dried *Potamogeton crispus*	26.8		[110]
	Alkali pretreated dried *Potamogeton crispus*	44.2		[110]
Methylene blue dye	Microwave-HAp	33.3		[111]
	MnO2-loaded biochar	248.96		[112]
Congo red dye	HAp-CS	769	pH 2–10	[113]
	Zinc peroxide (ZnO2) nanomaterial	208		[114]
Tetracycline	Zinc (II)-modified hydroxyapatites	168.5	298 K, pH 5	[102]
	Mn-N-doped carbon aerogels (MCA)	917.2	pH 4	[115]

**Table 4:** Comparison of the adsorption capacities of nano HAp and its composites with other absorbents on fluoride ions.

Adsorbate	Adsorbents	Defluoridation Capacity(mg F^−^ kg^−1^)	Conditions	Refs.
	Nanohydroxyapatite	1296	T 303 K	[129]
	Nanohydroxyapatite/chitin composite	2840	T 303 K	[129]
	Nanohydroxyapatite	1845	T 303 K, pH 3	[130]
	Alginate bioencapsulated nanohydroxyapatite composite	3870	T 303 K	[131]
	Nanohydroxyapatite in gelatin polymatrix	4157	T 303 K, pH 5	[132]
	Marble apatite (synthesized using ultrasonication method)	1826	T 303 K, pH 7	[142]
	Marble apatite (synthesized using conventional method)	960	T 303 K, pH 7	[142]
	Imidazolium ionic liquid modified chitosan	8.068		[143]
	Alginate beads modified with functionalized silica particles	51.02		[144]

## Data Availability

Data sharing is not applicable to this review.
